# 细胞表面糖蛋白相互作用规模化解析新技术及应用

**DOI:** 10.3724/SP.J.1123.2025.04001

**Published:** 2026-05-08

**Authors:** Yi LIU, Ci WU, Qun ZHAO, Zhen LIANG, Fuyun ZHANG, Lihua ZHANG

**Affiliations:** 1.大连海洋大学，辽宁 大连 116023; 1. Dalian Ocean University，Dalian 116023，China; 2.医学蛋白质组全国重点实验室，中国科学院大连化学物理研究所，辽宁 大连 116023; 2. State Key Laboratory of Medical Proteomics，Dalian Institute of Chemical Physics，Chinese Academy of Sciences，Dalian 116023，China; 3.辽宁师范大学，辽宁 大连 116029; 3. Liaoning Normal University，Dalian 116029，China

**Keywords:** 原位化学交联, 糖蛋白相互作用, 选择性富集, 液相色谱-串联质谱, *in vivo* chemical crosslinking, glycoprotein interactions, selective enrichment, liquid chromatography-tandem mass spectrometry （LC-MS/MS）

## Abstract

细胞表面糖蛋白复合物通过动态糖基化修饰调控细胞间识别、信号转导和免疫调节等关键生物学过程，其具有时空特异性的原位相互作用网络直接影响疾病的发生与治疗响应。然而，由于细胞表面糖蛋白相互作用通常具有弱结合力、瞬时性及糖基化修饰的复杂性，研究这类相互作用面临诸多技术性挑战。在本研究中，采用不可透膜型交联剂，结合氧化糖链-酰肼特异性反应与原位化学交联-质谱技术（CX-MS），成功实现了活细胞表面糖蛋白相互作用的高选择性捕获。该方法在人宫颈癌细胞（HeLa细胞）中共鉴定出4 457个高置信度糖基化位点，对应1 637个糖蛋白，其中84%的糖蛋白定位于细胞表面或细胞外相关分泌途径。进一步分析发现，在鉴定的蛋白-蛋白相互作用网络中，90%以上为糖蛋白-糖蛋白相互作用、糖蛋白-关联蛋白相互作用及关联蛋白间的相互作用网络，其中包括整合素β1（ITGB1）等关键糖蛋白介导的相互作用。该方法为深入解析细胞表面糖蛋白相互作用中的糖链功能提供了重要依据，有助于揭示糖基化在细胞信号传导与免疫调控等生物学过程中的关键作用，并为疾病的早期诊断和精准治疗提供新的策略。

## Abstract (en)

Cell surface glycoprotein complexes regulate critical biological processes such as intercellular recognition， signal transduction， and immune modulation through dynamic glycosylation modifications. Their spatiotemporally specific *in vivo* interaction networks directly influence disease pathogenesis and therapeutic responses. However， studying these interactions faces substantial technical challenges due to the weak/transient nature of glycoprotein interactions and the complexity of glycosylation modifications. In this study， we employed membrane-impermeable crosslinkers combined with oxidized glycan-hydrazide specific reactions and in vivo chemical crosslinking-mass spectrometry technology， successfully achieving high-selectivity capture of glycoprotein interactions on living cell surfaces. This approach identified 4 457 high-confidence glycosylation sites corresponding to 1 637 glycoproteins in HeLa cells， with 84% localized to the cell surface. Further analysis revealed that over 90% of the identified protein-protein interaction networks comprised glycoprotein-glycoprotein interactions， glycoprotein-associated protein interactions， and associated protein interaction networks. Key glycoprotein-mediated interactions， such as those involving integrin beta-1（ITGB1）， were elucidated. This methodology provides crucial insights for deciphering glycan functionality in cell surface glycoprotein interactions， contributes to revealing the pivotal role of glycosylation in biological processes like cellular signaling and immune regulation， and offers novel strategies for early disease diagnosis and precision therapeutics

细胞膜上的蛋白质是细胞间通讯和感知、响应外部环境信号的关键介质，通过与细胞外基质及细胞内蛋白的复杂互动，构建动态的相互作用网络，确保细胞能够精准响应微环境的变化^［1］^。糖基化修饰作为细胞表面蛋白的重要特征之一，其糖链在细胞间识别、通信和细胞黏附等多种生物活动中发挥重要作用^［2］^。异常糖基化可能影响细胞膜蛋白之间的相互作用，并与多种疾病的发生发展密切相关。例如，肿瘤微环境中唾液酸聚糖与肿瘤浸润免疫细胞上唾液酸结合免疫球蛋白样凝集素（Siglecs）受体之间的相互作用，可能成为预防和治疗多种疾病免疫调节的新潜在靶标^［3-5］^。因此，深入研究细胞表面糖蛋白的相互作用网络，对于理解特定时空下的细胞信号调控机制以及发现关键药物靶点至关重要。然而，此类研究面临巨大技术挑战^［6］^，主要源于相互作用的弱结合力、瞬时性、糖基化表达丰度低以及糖链结构的高度复杂性。

目前，细胞表面糖蛋白相互作用的研究方法主要包括基于凝集素亲和纯化法和化学交联质谱法。亲和纯化-质谱（AP-MS）方法，利用凝集素（lectin）或特异性糖链抗体识别糖蛋白，并结合质谱分析进行高通量鉴定^［7］^。然而，AP-MS仅能在细胞裂解液中分析蛋白相互作用，难以检测到细胞表面糖蛋白的短暂或弱相互作用，且这些膜蛋白需要在表面活性剂和高盐浓度等严苛条件下溶解，相互作用极易被破坏。进而，基于化学交联的细胞表面糖蛋白原位交联技术被提出，具有捕获弱/瞬时相互作用、分析灵敏度高和通量高等优点，已成为糖蛋白相互作用分析领域的研究热点^［8-10］^。由于糖链中缺乏特异性可交联基团，研究者多通过使用含有反应活性基团（如光交联基团或者叠氮基团）的糖类似物，选择性标记细胞表面的特定糖配基，然后结合交联技术实现糖蛋白相互作用的原位捕获与分析^［11，12］^。Kohler等^［13］^采用糖代谢标记法，将双吖丙啶光反应活性基团引入细胞表面糖链，利用紫外光激活该基团与细胞外糖结合蛋白（GBP）之间发生共价交联。通过向细胞中加入生物素化的GBP半乳糖凝集素-1，并结合亲和纯化和质谱技术对交联复合物进行分析，他们成功鉴定出癌胚抗原CEA、层粘连蛋白、整合素β1（integrin beta-1， ITGB1）和分化簇44（cluster of differentiation 44， CD44）等85 种半乳糖凝集素-1配体。然而，以上代谢标记技术通常依赖复杂的基因改造技术，成本较高，需要引入特定类型的代谢标记分子。此外，所采用的糖类似物中的功能团可能会干扰糖蛋白与蛋白之间的相互作用。

为了开发规模化的细胞表面糖蛋白原位交联策略，Wu等^［14］^采用了不透膜化学交联剂二（磺基琥珀酰亚胺）辛二酸酯（BS^3^）用于原位固定细胞膜表面蛋白之间的相互作用，随后通过糖基化蛋白的酰肼富集技术，选择性地富集细胞表面糖基化蛋白，从而显著提高了细胞表面糖蛋白组的解析能力。然而，糖蛋白之间的相互作用仍需进一步深入研究。在这一过程中，研究者面临两个主要挑战：一方面，大多数与糖蛋白相互作用的蛋白为细胞表面蛋白，这些相互作用容易受到细胞内蛋白交联的干扰；另一方面，糖蛋白本身具有糖基化修饰的复杂性和低丰度特性，导致糖蛋白相互作用的捕获和质谱分析变得更加困难。因此，克服这些挑战对于揭示糖蛋白动态相互作用机制至关重要^［15］^。

为了实现细胞表面糖蛋白相互作用网络的高效原位捕获与规模化鉴定分析，本文聚焦于提高细胞表面糖蛋白相互作用的原位高效捕获和交联位点鉴定的覆盖度，发展了细胞表面蛋白相互作用鉴定的新技术。采用前期开发的不透膜且能够高效富集交联肽段的2，6-二甲基哌啶二磺基琥珀酰亚胺十三酸酯（DPSST）交联剂，利用其优异的富集效率和细胞膜不透过性，将化学交联技术与基于酰肼化学的糖蛋白富集技术结合，对活细胞表面糖蛋白原位相互作用进行高选择性捕获，结合纳升液相色谱-串联质谱联用技术，实现了活细胞表面糖蛋白相互作用和交联位点的深度覆盖分析。该技术的应用使得细胞表面糖蛋白相互作用网络的规模化解析成为可能，为深入理解糖蛋白在细胞中的分子作用机制提供了重要的技术工具。

## 1 实验部分

### 1.1 仪器、试剂与材料

Easy-nano LC 1200液相色谱系统结合Orbitrap Fusion Lumos质谱系统、Scientz-IID超声波细胞破碎仪、BWS-5恒温水浴锅、H5000-HC-E振荡反应器、SpeedVac SPD140DDA-230真空浓缩仪、HERAcell 150i细胞培养箱、Multiskan Go全波长酶标仪和nano Drop one微量分光光度计均购自Thermo Fisher Scientific公司；1-14K低温离心机购自Sigma（德国）；IMS-50全自动制冰机购自常熟雪科电器；1290液相色谱仪购自安捷伦公司。

7-氨基-碳十三-二酸二甲酯、2，6-二甲基哌啶购自西安都创科技有限公司；*N*-羟基琥珀酰亚胺（NHS）、溴乙酸、1-（3-二甲氨基丙基）-3-乙基碳二亚胺盐酸盐（EDCI）、三乙胺（TEA）购自百灵威科技有限公司；二甲基亚砜（DMSO）、*N，N*-二甲基甲酰胺（DMF）、1-羟基苯并三唑（HOBt）、3-氨基丙酸、*N，N*-二异丙基乙胺（DIEA）、*N*-羟基琥珀酰亚胺磺酸钠购自梯希爱公司（日本）；碳酸氢铵（ABC）、高碘酸钠、十二烷基磺酸钠（SDS）、氨水、三乙基碳酸氢铵缓冲液（TEAB）、二硫苏糖醇（DTT）、碘乙酰胺（IAA）、甲酸（FA）、三氟乙酸（TFA）、尿素、蛋白酶抑制剂购自Sigma-Aldrich（美国）；氯化钠、醋酸、醋酸钠购自科密欧；测序级胰蛋白酶购自Promega（美国）；PNGase F酶购自纽英伦生物技术公司，4-羟乙基哌嗪乙磺酸（HEPES）缓冲液、杜氏改良Eagle（DMEM）培养基、磷酸盐缓冲液（PBS，pH=7.4）、三乙醇胺缓冲盐水溶液（TBS，20×）、固定化抗TMT抗体树脂（简称TMT抗体树脂）、酰肼微球（UltraLink^TM^酰肼树脂）购自Thermo Fisher Scientific（美国）；BCA蛋白浓度试剂盒购自碧云天生物公司；氯化-1-十二烷基-3-甲基咪唑（C12Im-Cl）离子液体购自成捷化学；色谱级乙腈和甲醇购自Merck（德国）；去离子水经Milli-Q超纯水系统（Millipore，美国）制备。高pH值反相色谱柱及C18反相分离柱（15 cm×150 μm， 1.9 μm）为实验室自行填装。

### 1.2 交联剂的制备

将3-溴丙酸滴加到氢氧化钠水溶液中冰浴搅拌，取2，6-二甲基哌啶滴加至上述混合液中，搅拌3 d。加浓盐酸将溶液pH调为3.0。利用二氯甲烷萃取产物，旋转蒸发除去有机相，得到2，6-二甲基哌啶基乙酸；将化合物2，6-二甲基哌啶基乙酸​溶于无水DMSO中，依次加入NHS和EDCI，室温下搅拌使其充分溶解，25 ℃反应过夜，即得到2，6-二甲基哌啶基乙酸琥珀酰亚胺酯中间体；继续向反应液中加入TEA及3-氨基丙酸，25 ℃反应30 min，经柱层析纯化，得到2，6-二甲基哌啶基乙酰丙酸；将所得的2，6-二甲基哌啶基乙酰丙酸​溶于无水DMF 中，依次加入7-氨基丙二酸二甲酯盐酸盐、EDCI、HOBt​ 及过量的 DIEA，于室温下搅拌反应15 h，经分离纯化得到2，6-二甲基哌啶基庚二甲酯，后续在碱性条件下水解，加盐酸调节溶液pH至酸性，制得2，6-二甲基哌啶基庚二酸；再加入*N*-羟基琥珀酰亚胺磺酸盐和EDCI，25 ℃反应24 h，制得2，6-二甲基哌啶二磺基琥珀酰亚胺十三酸酯（即DPSST交联剂）。

### 1.3 细胞培养

人宫颈癌细胞（HeLa细胞）在37℃、5% CO₂的恒温培养箱中，采用添加了10%（体积分数）胎牛血清（FBS）和1%（体积分数）青霉素-链霉素双抗溶液的高糖DMEM培养基进行贴壁培养。当细胞融合度达到70%~80%时，收集细胞用于后续实验。

### 1.4 细胞原位交联

收集HeLa细胞，计数1.5×10⁸ 个，用PBS洗涤3 次，去除残留的培养基，4 ℃下以500 g离心5 min，收集细胞沉淀。将DPSST交联剂溶于PBS中，配制成5 mmol/L的工作液，缓慢加入至细胞沉淀中，加入交联液体积为细胞沉淀体积的4~5倍。轻轻吹散细胞，确保均匀分散。于室温下轻轻摇晃细胞悬液，进行交联反应10 min。反应结束后，4 ℃下以500 g离心细胞，弃去交联液。接着，加入等体积的50 mmol/L ABC于细胞中，室温反应10 min，进行淬灭处理，随后在4 ℃、500 g条件下离心得到交联后的细胞沉淀。

### 1.5 细胞表面N-连接糖蛋白的氧化

配制氧化缓冲液：在去离子水中加入氯化钠和醋酸钠，配制成终浓度为150 mmol/L的氯化钠溶液和100 mmol/L的醋酸钠溶液，缓慢加入醋酸调节pH至5.5。然后，将NaIO_4_加入到配制好的氧化缓冲液中，得到终浓度为1 mmol/L的氧化液，用于细胞氧化。将与交联液等体积的氧化液加入到交联后的HeLa细胞沉淀中，轻轻吹散细胞，4 ℃下缓慢旋转氧化反应1 h后，4 ℃下以500 g离心5 min，收集氧化后的细胞沉淀。

### 1.6 酰肼法富集糖蛋白

配制细胞裂解液：2% SDS、1%蛋白酶抑制剂及0.1%离子液体溶解于氧化缓冲液中。将HeLa细胞沉淀重悬于细胞裂解液中，超声破碎至无色透明澄清液体（破碎条件：80 W，开5 s，关5 s，冰浴3 min）。采用BCA法测定细胞裂解液中的蛋白浓度，每1×10^7^ 细胞量裂解液加入1 mL酰肼微球，取1 mL充分混匀的酰肼微球悬液，加入至1×10⁷个细胞所对应的裂解液​中，于室温下温和孵育12 h，进行糖肽富集。以2 000 g离心5 min去除上清液，将8 mol/L尿素、0.4 mol/L TEAB和0.1% SDS溶于水中，配制成洗涤缓冲液，洗涤酰肼微球3次，每次5 min， 2 000 g离心5 min，以去除非特异性吸附。

### 1.7 交联糖蛋白样品预处理

向酰肼微球中加入1 mL 10 mmol/L 的 DTT 溶液（溶剂为 50 mmol/L HEPES， pH 8.0），室温孵育30 min以还原蛋白。使用50 mmol/L HEPES溶液1 mL清洗酰肼微球，随后加入1 mL 10 mmol/L的 DTT溶液（溶剂为50 mmol/L HEPES， pH 8.0）室温避光孵育30 min进行烷基化。向酰肼微球中加入8 mol/L尿素/0.4 mol/L TEAB缓冲液1 mL，洗涤4 次，每次5 min，以去除非特异性结合蛋白。将酰肼微球重悬于TBS缓冲液中，加入胰蛋白酶（酶与蛋白样品的质量比为1∶50），37 ℃酶解12 h。补加等量的胰蛋白酶，继续在37 ℃下酶解4 h。酶解后，收集上清液（内含DPSST交联肽段）。

接着向酰肼微球中加入1 μL PNGase F酶，37 ℃酶解12 h。酰肼微球经含0.1%（体积分数）FA、80%（体积分数）ACN水溶液​洗脱2次。每次1 mL，合并洗脱样品，冻干，得到糖基化肽段。

### 1.8 交联肽段选择性富集

预处理TMT抗体树脂：用2 倍体积的TBS缓冲液洗涤2 次，每次缓慢摆动5 min，2 000 g离心5 min。将酶解上清液加入到TMT抗体树脂中，混合均匀，4 ℃旋转孵育12 h，2 000 g离心5 min。去掉上清液，分别用2倍体积的TBS缓冲液洗涤TMT 抗体树脂4次，并用去离子水（pH=7）洗涤4 次，每次5 min，以去除非特异性吸附。向TMT抗体树脂中加入500 μL 50% ACN水溶液（含0.4% TFA，均为体积分数）洗脱液，室温孵育30 min，2 000 g离心5 min，收集上清液。重复洗脱步骤2次，合并洗脱液后冻干得到富集交联肽段。

### 1.9 肽段除盐与分级

为提高质谱对富集交联肽段的鉴定灵敏度，采用高pH C18反相柱对交联富集肽段样品以及糖蛋白样品分别进行分级。色谱条件：实验室自制C18反相色谱柱（15 cm×2.3 mm，5 µm），检测波长为214 nm，柱温为室温，流速0.25 mL/min。流动相A为2%（体积分数）ACN水溶液，B为98%（体积分数）ACN水溶液，二者均用氨水调pH为10。梯度洗脱程序：0~10 min，100%A；10.1~45 min，98%A~70%A；45~60 min，70%A~55%A；60~70 min，55%A~10%A；70~80 min，10%A。

样品冻干后，复溶于0.1%（体积分数）FA水溶液中，待nano LC-MS/MS数据采集。

### 1.10 nano LC-MS/MS分析

采用Easy-nano LC 1200色谱系统联用Orbitrap Fusion Lumos质谱仪，对交联肽段和糖基化肽段分别进行分析。两种样品均使用C18反相分离柱（15 cm×150 μm）进行分离，内部填料为ReproSil-Pur C18-AQ硅球（1.9 µm， 12 nm）。液相色谱条件如下：A相为0.1% FA水溶液，B相为含0.1% FA的80% ACN水溶液，流速为600 nL/min。采用120 min梯度（0~38 min，7%B~18%B；38~84 min，18%B~35%B；84~112 min，35%B~50%B；112~113 min，50%B~95%B；113~120 min，95%B）。均采用数据依赖型质谱采集方法（data-dependent acquisition， DDA），设置如下：一级扫描范围350~1 500，分辨率60 000（*m/z*=200），RF Lens为30%，二级扫描最小*m/z*为110，分辨率为15 000（*m/z*=200），母离子隔离窗口为*m/z* 1.6，一级和二级扫描的自动增益控制（AGC）分别为400 000和50 000，最大注射时间分别为50 ms和30 ms。强度大于20 000的前20 个母离子进行高能碰撞解离（HCD）碎裂。动态排除时间设置为20 s，归一化的HCD碰撞能量设为35%，其中交联肽段样品选择价态3~8 价，糖基化肽段样品选择价态2~8 价。

### 1.11 数据分析

交联肽段样品数据分析：交联肽段及交联位点信息的鉴定采用pLink 2.0软件（版本2.3.9）进行^［16］^。搜库参数设置如下：母离子和碎片离子质量相对偏差容限为20×10⁻⁶，母离子质量筛选相对偏差容限为10×10⁻⁶；赖氨酸（K）和蛋白N端设置为交联反应位点，选用胰蛋白酶，最多3 个漏切位点。肽段的长度范围是5~60 个，肽段的分子质量范围是500~6 000 Da。半胱氨酸（C）的脲甲基化为固定修饰，蛋白N端乙酰化和甲硫氨酸（M）的氧化为可变修饰。在≤1%的错误发现率（FDR）下对肽段谱图匹配（PSMs）进行过滤。交联肽段的质量偏移为447.31，单端交联肽段质量偏移为465.32。

糖基化位点的鉴定：将质谱生成的原始（*.Raw）文件利用MaxQuant（v1.4.0.5）软件进行搜库，数据库为uniprot human（人源蛋白组，包括20 422个蛋白，下载时间2023年4月）。数据库在搜索前会被搜库软件自动转换成正-反库的形式。具体的搜库参数如下：酶切类型设为胰蛋白酶的特异性酶切；允许最多两个漏切位点；半胱氨酸的烷基化（+57.021 5 Da）设为固定化修饰；甲硫氨酸的氧化（+15.994 9 Da）、蛋白的N端乙酰化（+42.010 6 Da）和天冬酰胺的脱酰氨基（+0.984 0 Da）设为可变修饰。

## 2 结果与讨论

### 2.1 基于原位交联-糖链富集的细胞表面蛋白相互作用组鉴定方法建立

细胞表面蛋白质的糖基化修饰作为细胞与外界环境之间的重要信号，发挥着关键作用。近年来，原位化学交联技术的发展通过交联固定细胞表面蛋白质的相互作用，结合亲和纯化的方法，能够有效避免在细胞裂解和富集纯化过程中蛋白质间弱相互作用的丢失，同时可提供蛋白质相互作用的界面关系^［17］^。基于这一策略，为了构建细胞表面糖蛋白质相互作用网络，本论文发展了一种将细胞表面蛋白质原位交联与糖基化特异性捕获技术融合，并结合糖基化交联肽段一步法富集的策略，用于细胞表面糖蛋白相互作用的深度解析，具体流程如图1a所示。首先，利用研究团队开发的新型靶向细胞表面的交联剂DPSST（如图1b）进行原位交联。该交联剂包含3个功能基团，一端含有2，6-二甲基哌啶的可富集基团，两端分别含有*N*-羟基琥珀酰亚胺磺酸基团，能够与蛋白质的赖氨酸残基末端或N端氨基发生酰胺化反应，从而在蛋白质复合物中实现空间邻近赖氨酸的化学交联。同时，二甲基哌啶基团的引入使得该交联剂能够通过“一步法”实现选择性富集，进而在活细胞内实现交联位点的特异性捕获。

**图1:**
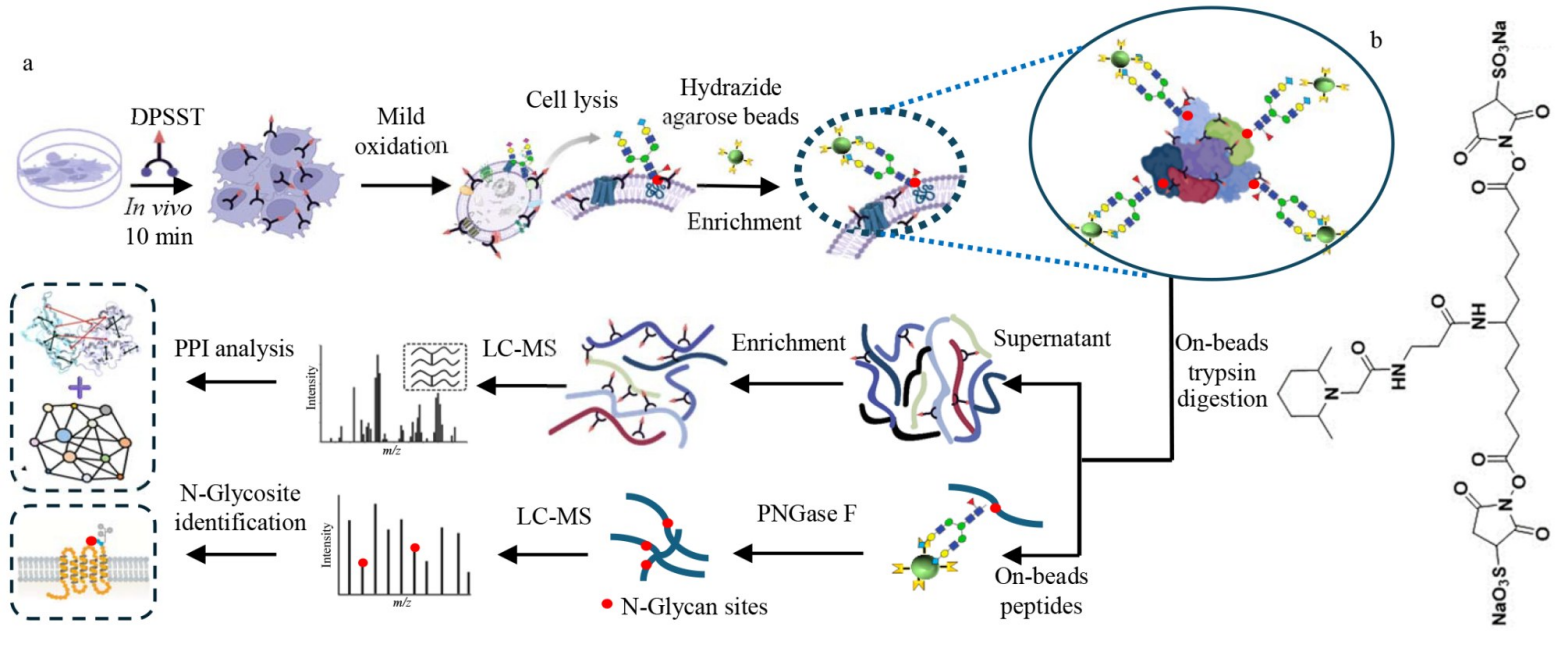
（a）细胞表面糖蛋白相互作用分析实验流程和（b）交联剂DPSST的结构式

其次，采用低浓度高碘酸钠氧化法将细胞表面唾液酸糖链转化为醛基，并利用酰肼微球特异性捕获细胞表面的糖蛋白复合物。使用胰蛋白酶对酰肼微球捕获的糖蛋白复合物进行原位酶解，并通过TMT抗体树脂进行“一步法”选择性富集，实现交联肽段的鉴定。同时，采用PNGase F酶释放酰肼微球上的糖肽，以鉴定糖基化位点。数据采集采用nano LC-MS/MS技术，并利用pLink 2.0交联搜索软件及生物信息学技术对细胞表面糖蛋白复合物的空间相互作用信息进行深入解析。

### 2.2 细胞表面糖蛋白与糖基化位点的分析

通过酰肼化学富集策略与质谱联用技术，成功实现了细胞表面糖蛋白的规模化解析（图2a）。实验共鉴定到4 457个高置信度糖基化位点，覆盖1 637种糖蛋白，其中单糖基化修饰位点774 个（47.3%），多个（>2）糖基化修饰位点863个（52.7%），揭示了细胞表面糖基化修饰的广泛性与复杂性。基因本体论（gene ontology， GO）系统性功能注释表明，84%的糖蛋白定位于细胞表面或细胞外相关分泌途径，且43%的蛋白明确富集于质膜（plasma membrane）（图2b），凸显了糖基化修饰在膜蛋白稳态调控中的核心作用。

**图2:**
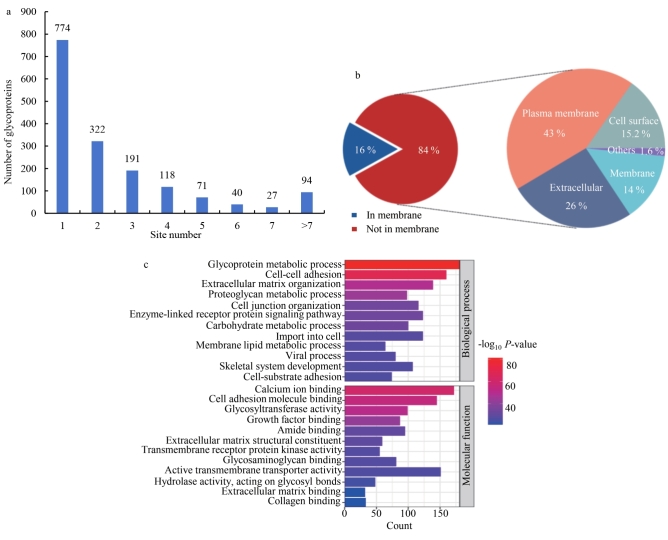
（a） HeLa细胞表面糖蛋白上的糖基化位点分布图；（b）细胞组成；（c）生物过程和分子功能

进一步的生物过程及功能分析显示（图2c），这些糖蛋白广泛参与细胞外基质动态组装、跨膜受体介导的信号转导（如Wnt、Notch通路）、糖蛋白代谢调控以及膜脂代谢与膜筏微区动态调控等生物学过程，系统调控基底膜重构、机械信号感知、糖基化修饰时空特异性及膜流动性等细胞活动。该研究系统绘制了细胞表面糖蛋白的功能图谱，为解析糖基化修饰介导的跨膜信号网络及开发靶向糖蛋白的诊疗策略提供了重要资源。

所鉴定的糖蛋白主要参与细胞黏附分子结合、细胞外基质结合、糖基转移酶活性、跨膜受体酪氨酸激酶活性及糖苷键水解酶活性等分子功能，涉及细胞黏附识别、糖链修饰、信号启动与糖链重塑等过程。其中，CD9、CD44、表皮生长因子受体和整合素亚基（如ITGB1）等关键糖蛋白在信号传导、肿瘤转移、免疫突触形成及病原体侵染等病理生理事件中发挥重要作用。

### 2.3 细胞表面糖蛋白相互作用原位解析

采用pLink 2.0对得到的交联数据进行解析，共计鉴定到涉及120个蛋白质的274对分子内交联位点。为了考察交联位点的准确性，首先将120 个蛋白质匹配至Protein Data Bank（PDB）数据库的蛋白结构中，并对鉴定到的交联位点距离进行测量。根据274对蛋白内交联位点的空间距离统计，发现94.9%的位点间距均在DPSST交联剂的臂长约束（35.8 Å）范围内（图3a），充分证实了该方法在蛋白三维构象解析中的可靠性（图3b）。接着，对鉴定到的蛋白-蛋白相互作用（PPIs）进行了可信度评估，通过STRING数据库的置信度评分验证，绘制了其得分分布图（图3c）。其中，23对相互作用关系评分≥0.7（高置信区间），98对未见报道的相互作用关系占比72.1%，相比STRING数据库中蛋白相互作用得分分布，本研究的蛋白相互作用鉴定组具有更高的可信度。

**图3:**
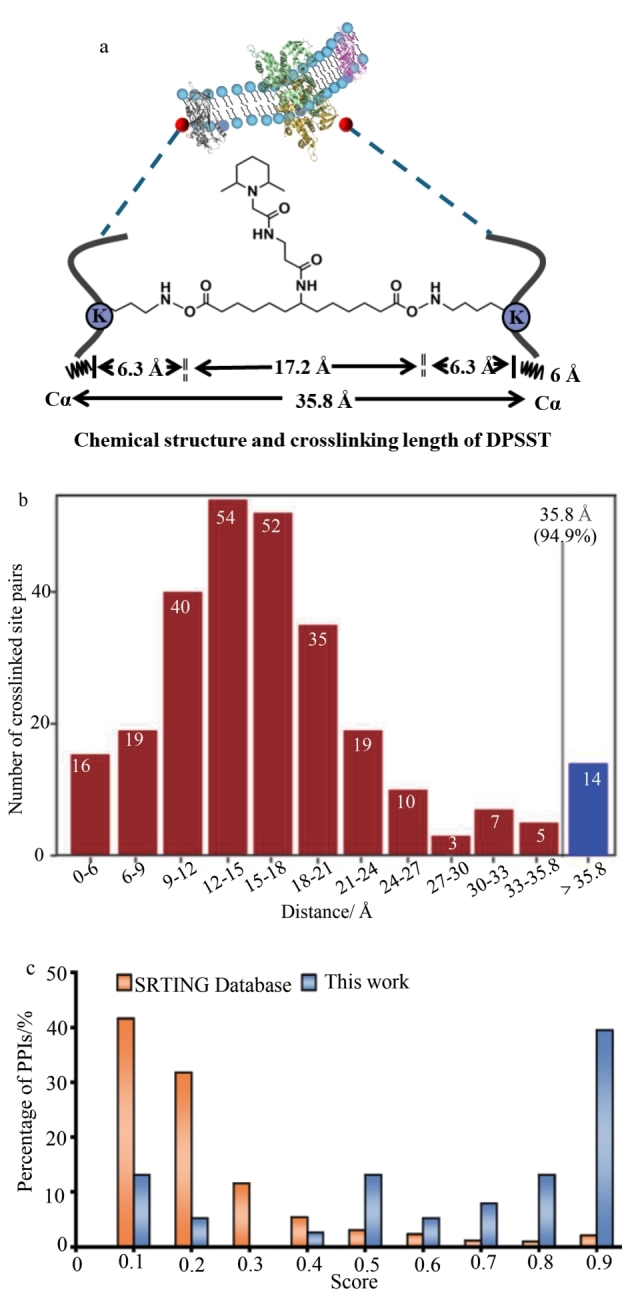
（a）交联剂DPSST的最大距离约束；（b）映射到PDB结构上的蛋白质内部交联的Cα-Cα原子间距离分布；（c）蛋白间相互作用置信度分析

利用化学交联技术系统解析生理条件下蛋白的动态构象，其中ITGB1共捕获到16 对高置信度蛋白内交联位点（图4a），所有位点均精确分布于交联剂的约束距离范围内。ITGB1是定位于细胞表面的糖蛋白，作为细胞-基质黏附和机械信号转导的关键介质，其构象动态与多种病理过程密切相关——例如，在肿瘤侵袭转移中，ITGB1通过调控整合素α/β异源二聚体的构象重排，激活下游黏着斑激酶（focal adhesion kinase， FAK）信号轴，从而促进细胞迁移、侵袭及细胞外基质降解。此外，本研究还鉴定到3对ITGB1与FBXO34的蛋白间交联位点。值得注意的是，FBXO34作为一类高度动态的F-box家族蛋白，其亚细胞定位尚未明确，且因富含柔性结构域而难以通过传统结构生物学方法解析。我们基于AlphaFold2的蛋白结构预测模型^［18］^，构建了FBXO34的拓扑框架，发现其N端F-box结构域与C端无序区域之间的交联位点可能参与泛素连接酶复合物的动态组装。为深入评估ITGB1蛋白N-糖基化位点与交联肽段的鉴定质量，进一步分析了ITGB1的二级质谱图特征（见图4b，4c）。可以看到，无论是糖肽谱图还是交联肽段谱图，α-肽段与β-肽段均产生连续且丰富的b/y离子，碎片离子覆盖度超过80%，且交联位点两侧肽段离子的质荷比测量值与理论值的相对偏差≤1×10⁻⁶。这些数据从谱图解析层面证实了交联肽段鉴定的高准确性，为后续蛋白构象建模及相互作用网络重构提供了坚实的技术保障。

**图4:**
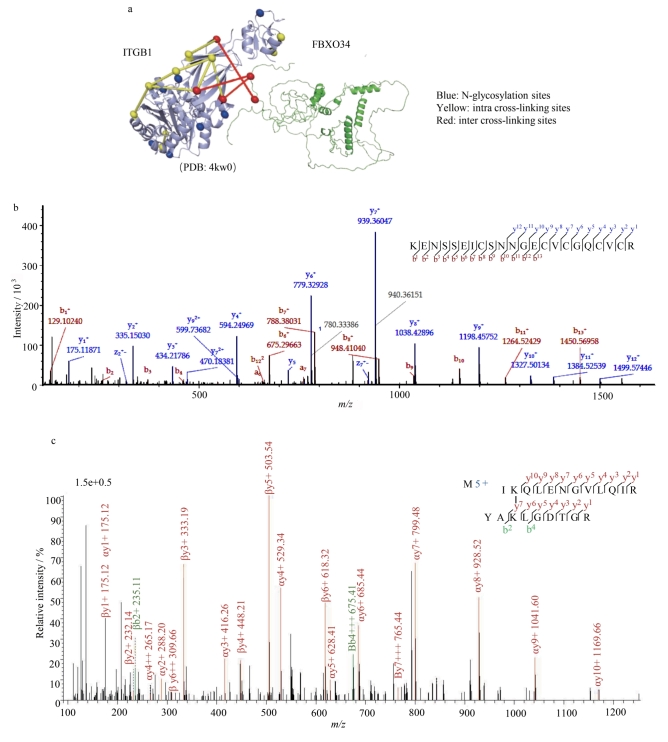
（a）交联信息匹配至ITGB1蛋白的高分辨率结构；（b）ITGB1蛋白N-糖基化位点鉴定谱图；（c）ITGB1蛋白交联肽段二级谱图

在相互作用网络方面，基于DPSST交联方法捕获的蛋白相互作用中，包含355 个交联位点，涉及95 个核心蛋白，形成136 对蛋白-蛋白相互作用网络。在这些相互作用中，糖蛋白占据了59 个位置（占总数的62.1%）。所绘制的相互作用网络中，高达90%的相互作用为糖蛋白-糖蛋白相互作用、糖蛋白-关联蛋白相互作用及关联蛋白间的延伸相互作用（见图5a），所鉴定到的细胞表面蛋白相互作用与离子通道、配体-受体、锚定蛋白、结合蛋白与酶等功能密切相关。这一结果充分证明了本研究所发展的策略在细胞表面糖蛋白相互作用分析中的高特异性。

**图5:**
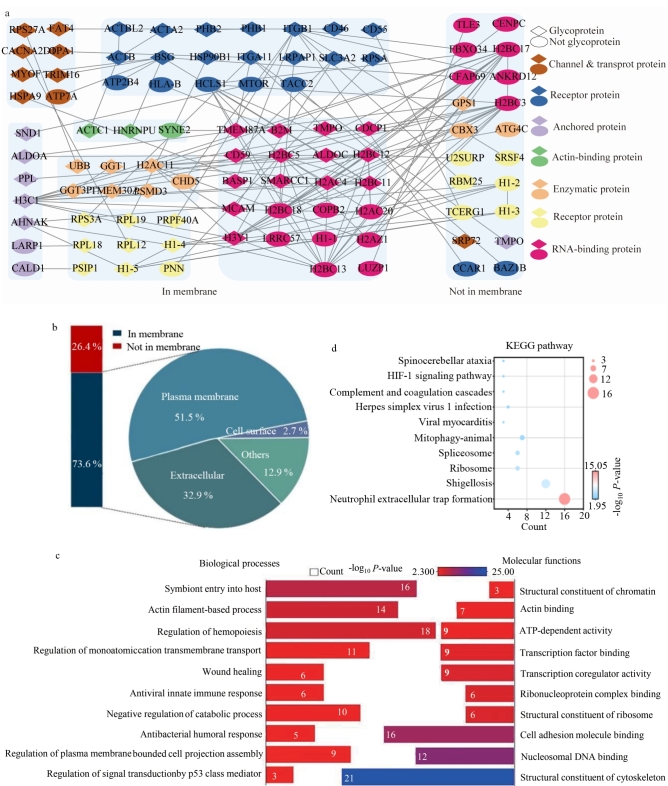
（a）95个蛋白的相互作用网络**；（**b）细胞组成；（c）生物过程和分子功能；（d）KEGG通路

进一步，对这些蛋白进行GO分析，发现鉴定到的交联蛋白在生物学功能上呈现多层次协同作用。细胞组成表明（图5b），这些蛋白显著富集于质膜、细胞外及细胞表面，占比达73.68%，提示大部分蛋白参与细胞蛋白稳态维持、应激及外部微环境传感。从生物过程（biological process）（图5c）可看出，核心功能集中于阳离子跨膜运输的动态调控（如钾离子、钠离子稳态）、介质信号转导级联反应以及细胞迁移与形态重塑，进一步体现了其在细胞通讯和应激响应中的枢纽作用。在分子功能层面，这些蛋白表现出与细胞黏附分子（如整合素、钙黏蛋白）的特异性结合活性，并作为细胞骨架的结构成分（如微管蛋白复合体、中间纤维）直接参与肌动蛋白（actin）聚合与应力纤维组装，从而调控细胞力学特性及运动能力。此外，KEGG通路富集分析（图5d）揭示了这些蛋白与中性粒细胞胞外诱捕网（NETs）形成及HIF-1信号通路等密切关联，综合上述结果，交联蛋白通过多维度功能整合，在免疫应答、能量代谢及病理微环境适应中发挥关键调控作用，为解析其分子机制提供了系统性视角。

## 3 结论

本工作开发了一种细胞表面糖蛋白相互作用分析新技术，通过细胞表面蛋白原位交联、糖基化-酰肼特异性反应与选择性捕获，以及糖基化交联肽段的“一步法”高效富集鉴定技术，实现了细胞表面糖蛋白的相互作用的规模化解析，为原位动态解析细胞表面糖蛋白介导的蛋白-蛋白相互作用网络提供了重要的技术工具。该方法直接在活细胞中进行，无需裂解细胞或固定处理，可完整保留糖蛋白在天然状态下的构象及瞬时动态相互作用，将不可透膜交联剂与特异性糖链标记技术联用，降低了非特异性结合风险，提高了数据的可靠性。将该方法用于HeLa细胞分析，富集到4 457个高置信度的糖基化位点，涉及1 637个糖蛋白，其中84%定位于细胞表面或细胞外相关分泌途径。交联位点的鉴定结果显示，在59个糖蛋白中（占相互作用节点总数的62.1%），90%的相互作用关系为糖蛋白与糖蛋白之间的直接相互作用、糖蛋白与关联蛋白的协同相互作用以及关联蛋白之间的延伸相互作用。这表明糖基化修饰可能通过多层级的相互作用架构来驱动网络的动态功能整合。此外，还揭示了糖蛋白（如ITGB1）在生理状态下的构象动态，其交联位点的分布与功能域（如配体结合区）高度吻合，为基于结构的药物设计提供了新的靶点。本研究建立的规模化分析方法不仅可揭示糖蛋白复合物的空间组织规律，更可为靶向糖基化修饰的药物设计、基于膜受体相互作用的疾病生物标志物发现提供分子机制层面的理论支撑，为突破传统蛋白相互作用研究在膜蛋白动态复合体解析中的技术瓶颈提供了新范式。
